# Epidemiological and Occupational Pattern of Patch-Test Reactions to p-Tert-butylphenol-formaldehyde Resin in North-Eastern Italy, 1997–2021

**DOI:** 10.3390/life15050698

**Published:** 2025-04-25

**Authors:** Luca Cegolon, Alessandro Badalini, Francesca Larese Filon

**Affiliations:** 1 Department of Medical, Surgical & Health Sciences, University of Trieste, 34128 Trieste, Italy; alessandro.badalini@asugi.sanita.fvg.it (A.B.); larese@units.it (F.L.F.); 2 Public Health Department, University Health Agency Giuliano-Isontina (ASUGI), 34128 Trieste, Italy; 3 Occupational Medicine Unit, University Health Agency Giuliano-Isontina (ASUGI), 34128 Trieste, Italy

**Keywords:** p-tert-butylphenol-formaldehyde resin (PTBP-FR), allergic contact dermatitis, sensitization, patch test, occupational exposure, clinical relevance, epidemiology

## Abstract

**Background**. Skin contact with items containing p-tert-butylphenol-formaldehyde resin (PTBP-FR) may induce sensitization and allergic contact dermatitis (ACD). **Methods**. This multi-centric cross-sectional study investigated the prevalence of sensitization to PTBP-FR in 30,629 consecutive outpatients patch-tested during 1997–2021 in four research centers from Northern Italy: Padua; Pordenone; Trieste; and Trento/Bolzano/Rovigo. Patch tests were applied on the upper back of patients with suspected ACD. All patches were removed after 48 h and read at 72 or 96 h. **Results**. The overall prevalence of PTBP-FR sensitization was 1.11% (=341/30,629) of cases, with lower prevalence occurring in the Province of Trento/Bolzano/Rovigo (0.36%). The body area most frequently affected were the hands (36.32%), followed by face (19.52%) and legs (8.09%). During 1997–2004, the prevalence of PTBP-FR positivity was significantly lower in Trento/Bolzano/Rovigo (aOR = 0.19; 95%CI: 0.11; 0.35), whereas it was higher among restaurant workers (aOR = 2.44; 95%CI: 1.44; 4.13). During the entire study period (1997–2021), excluding Trento/Bolzano/Rovigo, PTBP-FR positivity significantly decreased in the period 2011–2021 (aOR = 052; 95%CI: 0.39; 0.69) compared to 1997–2010 in males (OR = 0.69; 95%CI: 0.52; 0.91). **Conclusions**. Females were likely to react to PTBP-FR at patch tests. Prevalence of PTBP-FR sensitization significantly decreased over time, possibly reflecting reduced occupational and non-occupational exposure due to replacement of the resin with other adhesive products (acrylates or epoxy agents).

## 1. Introduction

P-tert-butylphenol-formaldehyde resin (PTBP-FR) is an alkyphenol resin synthesized by polymerization of para-tertiary-butylphenol (PTBP) and formaldehyde [1]. Thanks to its chemical and physical properties (heat resistance, durability, flexibility and rapid activity), PTBP-FR is widely employed to improve the function of poly-chloroprene adhesives [2] and as a glue to bond leather and rubber products [3]. PTBP-FR is applied and in shoes, hats, watchbands, belts, handbags, automobile interior upholstery, plywood, furniture, deodorants, diapers, nail adhesives, prostheses, dental bonding materials, disinfectants, insecticides, athletic tapes, poly-chloroprene adhesives and sauna shorts [1,3,4,5,6].

PTBP-FR is a hapten known to be able to induce sensitization, especially following skin contact with shoe adhesives, has been included in most standard patch-test series [7,8]. According to the main large databases, prevalence of PTBP-FR sensitization varied from 0.3% in Northern Ireland during 1987–1992 to 1.6% in Italy during 2007–2008, with estimates frequently being > 1% in the USA (Table 1) [9,10,11,12,13,14,15,16,17,18,19,20,21,22,23,24].

In North America prevalence of sensitization to PTBP-FR on 43,677 patients referred for patch testing from 2001 to 2018 was 7.1% among those with shoe contact dermatitis (CD) and 0.6% in those with hand CD (Table 1) [22].

Non-occupational allergic contact dermatitis (ACD) induced by PTBP-FR more commonly involves the feet [25], and several cases have been reported, mainly triggered by exposure to shoes [26,27,28,29]. For instance, in an investigation on 109 patients with foot CD and a shoe source of allergens from the North American Contact Dermatitis Group (NACDG) patch-tested during 2001–2004, PTBP-FR was the most common allergen—accounting for 24.7% positive patch-tests—and shoes were the source of the allergen [27]. In a subsequent NACDG investigation on 352 patients patch-tested during 2005–2018, with a shoe allergen source and foot as one of three sites of dermatitis, potassium dichromate was the most common allergen (29.8%), followed by PTBP-FR (20.1%) [28].

By contrast, the hands are more frequently involved in occupational ACD induced by PTBP-FR, especially among workers in shoe and leather industry [30], clothing manufacturing [31] and those exposed to paper sheets impregnated with resins [32]. The first case of ACD caused by PTBP-FR exposure was, in fact, reported by Malten in 1958 in a worker handling shoe adhesive [33].

Exposure to PTBP-FR can occur also through different routes. For instance, skin contact with Holter electrodes (3 M Red Dot 2239 ECG electrodes) caused ACD in three patients subsequently patch testing positive to PTBP-FR [34]. Likewise, a 46-year-old woman undergoing 24 h Holter examination developed vesicular dermatitis induced by PTBP-FR at each electrode site, one day after their removal from her chest [35]. A case of hand ACD persisting for about 5 years induced by PTBP-FR present in a basketball was reported in a 33-year-old non-atopic professional player [36]. Furthermore, a 56-year-old woman developed ACD on skin areas (neck and legs) directly in contact with her scuba wetsuit containing PTBP-FR adhesives [37].

In view of the above, this multi-centric cross-sectional study aimed to investigate prevalence of positive reactions in consecutive outpatients with suspected ACD who were patch- tested in Triveneto, an area of North-Eastern Italy.

## 2. Methods

Prevalence of sensitization to PTBP FR (1% in pet) was investigated in 30,629 consecutive patients undergoing patch test for suspected ACD during 1997–2021 (25 years) in four centers of Triveneto—Trieste, Padua, Pordenone and Bolzano/Trento/Rovigo—to identify potential trends and associated factors. This study was approved by the local ethical committee of Friuli Venezia Giulia (CEUR, protocol 092/2018, approval date 23 March 2018), and written informed consent was obtained from all participating patients.

### 2.1. Evaluation of Patients and Patch Testing

The clinical pattern of patients was assessed using the MOAHLFA Index (considering sex of patient, occupational dermatitis, atopic dermatitis, hand dermatitis, leg dermatitis, face dermatitis, age > 40 years) [38].

Occupation was classified using ISCO-88 codes and then summarized in groups with similar exposure.

Occupational dermatitis was assessed by a dermatologist or an occupational medicine consultant considering the clinical history, sites involved, occupational exposures and stop-and-go test.

All patients were patch-tested with Finn Chambers (Epitest, Tuusula, Finland) on Scanpor tape (Norgesplaster, Vennesla, Norway), using haptens produced by Chemotechnique Diagnostics (Vellinge, Sweden) and by FIRMA (Florence, Italy). During the overall period, the European baseline series and the extended Triveneto series (Supplementary Table S1) were used to patch test patients for suspected ACD. All patches were applied on the upper part of patients’ back and removed after 48 h. The area was examined upon removal of the patch (D2) and after 72/96 h (D3/D4), according to guidelines of the International Contact Dermatitis Research Group [39]:

Reaction degrees of +, ++ and +++ were considered positive.

Doubtful reactions (?+) were considered negative.

### 2.2. Statistical Analysis

Since Trento/Bolzano/Rovigo contributed only until 2004, prevalence of sensitization against PTBP-FR by explanatory variables was assessed in years 1997–2004 in all four research centers, whereas analysis across the entire study period (1997–2021) excluded Trento/Bolzano/Rovigo, since the latter center contributed only until 2004.

Continuous variables were presented as mean and standard deviation as well as median and interquartile range (IQR). Means were contrasted by t-test, medians by Mann-Whitney test, whereas the Chi-squared test was employed to compare categorical variables.

A backward stepwise procedure was used to build up two multivariable logistic regression models—Model 1 limited to years 1997–2004 yet including all centers and Model 2 stretching across the entire study period (1997–2021) but excluding Trento-Bolzano-Rovigo—to investigate the prevalence of sensitization to PTBP-FR, reporting adjusted odds ratios (aOR) with 95% confidence interval (95%CI).

The statistical analysis of the data was performed with STATA version 14.0 (Stata, College Station, TX, USA).

## 3. Results

Prevalence of PTBP-FR positivity was 1.35% (=206/15,307) for years 1997–2004 and 1.21% (328/26,996) across the entire study period (1997–2021) excluding Trento/Bolzano/Rovigo (Table 2). Considering all centers combined, the overall prevalence of PTBP-FR positivity was 1.11% (=341/30,629) during the entire study period (1997–2021) (Supplementary Table S2).

**Figure 1:**
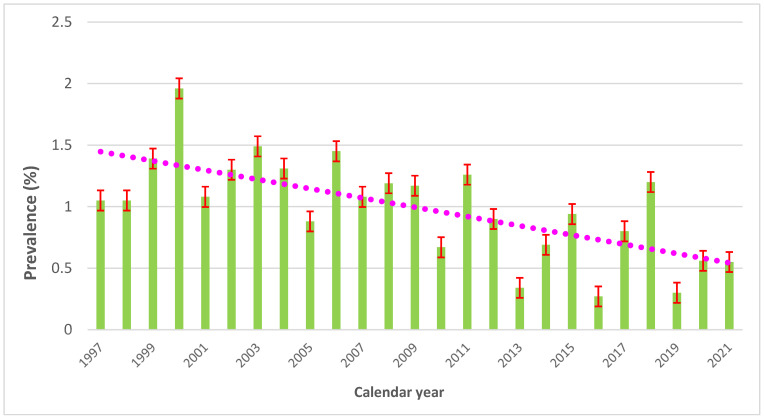
Yearly prevalence of positive patch tests to p-tert-butylphenol-formaldehyde resin (PTBP-FR) in North-Eastern Italy, 1997–2021 (all research centers combined), with a trendline (purple marked). Standard errors red-marked.

A variability of testing and sensitization was observed in the entire cohort, with a decreasing time trend from 1.96% in 2000 to 0.55% in 2021 (Supplementary Table S2 and Figure 1).

Table 2 displays the descriptive distribution of the study population by sensitization to PTBP-FR and explanatory factors, broken down by study period (1997–2004 across all centers vs. 1997–2021 excluding Trento/Bolzano/Rovigo). Supplementary Table S3 shows the distribution of patch tests executed and percentage of positive results by center and calendar period.

*Calendar Years 1997–2004*

As mentioned above, prevalence of PTBP-FR positivity during 1997–2004 was 1.35%, lower in Trento/Bolzano/Rovigo (0.36%) and higher in Pordenone (2.05%) and Padua (1.77%) (Table 2).

The median age of patients testing positive to PTBP-FR during 1997–2004 was 40 years, and sensitization was significantly higher in females (1.49%) than males (1.04%, *p* = 0.023). Prevalence of female sex among patients sensitized to PTBP-FR in 1997–2004 was 74.76% (=154/206).

The body area most frequently affected by contact dermatitis during 1997–2004 were the hands (41.23%), followed by face (16.08%) and legs (7.19%), with significantly higher prevalence in patients without hand involvement (1.73% vs. 1.19%, *p* = 0.020) (Table 2).

Prevalence of atopic dermatitis was 558 (4.52%) among patients undergoing patch testing and 2.43% (=5/206) among those testing positive to PTBP- FR. Prevalence of OCD was 8.71% (=1.334/15,307) among patients undergoing patch testing and 6.80% (=14/206) among those testing positive to PTBP-FR.

Prevalence of PTBP-FR sensitization was higher among restaurant workers (3.17%), leather artisans (3.06%) and workers of the chemistry industry (2.40%) (Table 2).

Table 3 displays multiple logistic regression analysis on factors associated with sensitization to PTBP-FR.

Prevalence of PTBP-FR sensitization was significantly lower in Trento/Bolzano/Rovigo (aOR = 0.19; 95%CI: 0.11; 0.35), among males (aOR = 0.69; 95%CI: 0.52; 0.91) and in other occupational categories (aOR = 0.60; 95%CI: 0.39; 0.91).

*Calendar years 1997–2021*

As mentioned above, prevalence of PTBP-FR positivity during 1997–2021 was 1.21%, slightly lower in Trieste (1.08%) compared to Padua (1.29%) or Pordenone (1.29%) (Table 2). Sensitization was significantly (*p* < 0.001) lower in years 2010–2021 (0.73%) compared to 1997–2010 (1.44%) and in males (0.97%) compared to females (1.38%). Prevalence of female sex among patients sensitized to PTBP-FR in 1997–2021 was 76.52% (=251/328).

Prevalence of atopic dermatitis was 10.48% (=2664/26,996) among patients undergoing patch testing and 8.54% (=28/328) among those testing positive to PTBP- FR. Prevalence of OCD was 7.76% (=2092/26,996) among patients undergoing patch testing and 8.84%% (=29/328) among those testing positive to PTPB-FR.

In terms of occupation, sensitization to PTBP-FR was significantly higher in general cleaners (2.25%), construction cleaners (5.88%) or hairdressers (2.12%) (Table 2).

At multiple regression analysis showed that, during 1997–2004 (Model 1), sensitization to PTBP-FR was significantly lower in Trento/Bolzano/Rovigo (aOR = 0.19; 95%CI: 0.11; 0.35) and higher among restaurant workers (aOR = 2.44; 95%CI: 1.44; 4.13). During the entire study period (1997–2021), excluding patients from Trento/Bolzano/Rovigo, prevalence of PTBP-FR sensitization was significantly lower in the period 2011–2021 (aOR = 0.52; 95%CI: 0.39; 0.69) compared to 1997–2010, among males (aOR = 0.69; 95%CI: 0.52; 0.91) and in other occupational categories (aOR = 0.60; 0.39; 0.91) (Table 3).

## 4. Discussion

### 4.1. Prevalence Sensitization to PTBP-FR

With variability by center and calendar year, prevalence of PTBP-FR sensitization was 1.35% during 1997–2004 compared to 1.21% across the entire study period (1997–2021), excluding Trento/Bolzano/Rovigo, progressively decreasing over time (Supplementary Table S2).

Excluding Trento/Bolzano/Rovigo, in patients patch-tested before 2010, prevalence of sensitization was 1.44%, decreasing to 0.73% afterwards, reaching the percentage of 0.55% in 2021. In Europe, prevalence of PTBP-FR sensitization remained rather steady over time, fluctuating from 0.59% (95%CI 0.41–07) during 2007–2010 to 0.50% (95%CI 0.43–0.58) in 2001–2014 [11], 0.54% (0.41–0.7) in 2015–2018 [12] and 0.47% (0.35–0.61) [13] in 2019–2020. Likewise, prevalence of sensitization to PTBP-RF was relatively stable over time in the USA, yet consistently > 1% [22].

In an earlier study from the University Hospital of Coimbra (Portugal) on 3106 patients (1391 males against 1715 females) patch-tested during 1982–1991 for suspected ACD, 74 patients were positive for PTBP-FR—a prevalence of 2.4% [10]. In another study on 1966 patients from the Netherlands with suspected ACD, 30 (1.5%) tested positive to PTBP-FR, which was confirmed as causative agent in 7 cases, whereas in six cases it was a probable causative factor and in 17 the etiological agent was unclear [11]. Likewise, patch-test positivity against PTBP-FR during 2009–2018 was 1.6% out of 441 patients with suspected shoe ACD in a clinical study using data from IVDK, a network of 58 dermatological departments from Germany, Switzerland, and Austria [4].

Prevalence of PTBP-FR sensitization can therefore be rather variable and influenced by several factors, including environmental conditions (humidity, high temperature), which could induce sweating and facilitate the release of the hapten from handworks [1,40,41,42,43,44,45], compliance with preventative measures (use of PPE and/or moisturizing creams); the modality of patch-test execution; variability of test reading [10,30,46,47]; and different concentrations of the main two allergens (dimers IX and X) in patch-test formulations [48]. An abnormal concentration of the latter dimers may in fact induce false negative results at patch test [8]. Information on the concentration of the latter main two allergens in patch-test preparations was not available in the present study.

### 4.2. Factors Associated with PTBP-FR Sensitization

In line with the literature, in the present study, the body area most frequently affected by CD in patients undergoing patch testing were the hands, followed by face and legs [10,37]. In the above study from the University Hospital of Coimbra (Portugal) on 3106 patients patch-tested during 1982–1991, in 67.57% (=50/74) cases, the feet were the most frequent site affected by dermatitis, due to shoes [10]. Since information on foot dermatitis was not available in the present study and leg dermatitis does not necessarily reflect foot involvement, prevalence of foot CD was inevitably even lower.

In the present study, the mean age of patients patch testing positive to PTBP-FR (42.5 years in 1997–2004 vs. 43.2 years in 1997–2021) was slightly higher than that reported elsewhere [11,49], but it was very much in line (43 years) with the above study from Portugal [10].

The majority of patients patch-tested in the present study were females (67.44% in 1997–2004 vs. 67.30% during the entire study period 1997–2021 excluding Trento/Bolzano/Rovigo), and patients testing positive to PTBP-FR were predominantly females both during 1997–2004 (74.76%) and across the entire study period excluding Trento/Bolzano/Rovigo (76.52%). Although the setting of the study does not allow us to infer causation, considering the over-representation of females among patients patch-tested for PTBP-FR for suspected ACD, 75.7% (=56/74) of patients positive to PTBP-FR among 3106 patch-tested for suspected ACD in Portugal during 1982–1991 were also females [10]. Furthermore, in the above study on 1966 patients from the Netherlands, 80% (=24/30) patch testing positive to PTBP-FR were females [11]. Likewise, female prevalence among 46 patients positive to PTBP-FR was 78% (=36/46) in a European study on 7094 patients (73% females) patch-tested for suspected ACD during 2005–2018 [8].

Moreover, multivariable logistic regression analysis showed that females were more likely to test positive across the entire study period, excluding Trento/Bolzano/Rovigo in the present investigation. Whilst sex-dependent predisposition may be involved, females are generally at risk of ACD due to a higher level of occupational and non-occupational exposure to allergens in their lifetime [50,51]. Moreover, female skin is generally thinner and more permeable to irritants and allergens [50]. However, shoe CD was more likely in males and patients younger than 40 years among 352 NACDG patients patch-tested during 2015–2018, subsequently diagnosed with foot ACD [28].

In the present study prevalence of atopic dermatitis among patients testing positive to PTBP-FR was 2.43% during 1997–2004, compared to 8.53% across the entire study period (1997–2021), in line with previous data from Triveneto [51].

### 4.3. The Impact of Occupation

Prevalence of occupational dermatitis among patients testing positive to PTBP-FR was 6.80% in 1997–2004 and, after excluding Trento/Bolzano/Rovigo, 8.84% across the entire study period (1997–2021).

Prevalence of occupational dermatitis was slightly higher (8.82%) among patients patch testing positive to PTBP-FR compared to those testing negative (8.23%). Higher rates (24.4%) of occupational exposure in patients testing positive to PTBP-FR were reported in an earlier investigation (before year 2000) on a selected population [52].

The progressive reduction of PTBP-FR sensitization over time observed in the present study—already reported in the open literature [49,53,54]—may reflect decreasing occupational exposure to the resin, mainly used in footwear, due to its progressive replacement with other glues and adhesive products (acrylates or epoxy agents) and/or higher compliance with health and safety at work (HSW) measures [49]. Although typically more allergenic than PTBP-FR, acrylates, not included in the patch-test formulation used in the present study, do not cross-react against PTBP-FR, hence, they cannot have determined false positive patch-test results.

For instance, a possible explanation for the lack of sensitization in maintenance activities within the industrial sectors (e.g., assemblers and technical maintenance staff) may reflect higher compliance with HSW measures and lower use of the resin for maintenance activities, although no information on this point was available. Likewise, the increased sensitization to PTBP-FR in restaurant workers found in the present study is hard to explain [1], since any worker can have skin contact with adhesive and glues during small maintenance activities or to leather shoes or neoprene gloves. Neoprene is a special synthetic rubber included in many products (e.g., wet suits, elastic supports, gloves, shoes, orthopaedic devices, adhesives, among other), which can contain PTBP-FR as an ingredient [6,55,56,57,58]. However, higher prevalence of sensitization to PTBP-FR in restaurant workers observed during 1997–2004—not confirmed across the entire study period (1997–2021)—may arguably reflect decreased occupational exposure over time.

The decreasing occupational exposure to PTBP-FR over time was also endorsed by the geographical distribution of sensitization, significantly lower in Trento/Bolzano/Rovigo (0.36%), at least during 1997–2004, and higher in Padua (1.29%) and Pordenone (1.30%). The areas of Pordenone and Padua are known to have a more developed industrial sectors, including leather factories in the Riviera del Brenta, just outside Padua city. By contrast, the economy of the provinces of Trento-Bolzano mainly relies on agriculture and tourism, Rovigo on agriculture and Trieste on tertiary sector, with limited industrial activities entailing potential occupational exposure to glues/adhesives [45]. Moreover, the provinces of Trentino and Bolzano are mountain areas featuring lower average temperatures (especially in winter) than the other three research areas-hence, different environmental conditions may account for the geographical differences in PTBP-FR sensitization. While leather shoes worn without socks in areas with warmer temperatures may contribute to inducing sensitization to PTBP-FR, lower environmental temperatures may inhibit the release of the latter hapten [1].

### 4.4. Strengths and Limitations

The present is the largest study investigating PTBP-FR patch-test positivity in Italy over a long time period (25 years), employing a multi-center data collection, while also assessing the impact of occupation and yielding adjusted prevalence estimates of PTBP-FR sensitization over time. Moreover, this study collected information on patient occupation, and information not normally available in the literature. This adds value to the present study.

Study limitations include the cross-sectional design, the variability of testing prevalence by research center and calendar year and the lack of relevance definition for PTBP-FR sensitization. In order to avoid potential interpretation bias, an assessment of clinical relevance of sensitizations was averted, as was also in other epidemiological studies [11,12,13,59,60]. Another limitation is the lack of data on foot involvement, since PTBP-FR is primarily considered a shoe allergen. However, most epidemiological studies from Europe and North America considered hands, legs and face as body sites.

## 5. Conclusions

The overall prevalence of PTBP-FR sensitization was 1.11% during 1997–2021 (including all centers combined), significantly decreasing over time, likely reflecting a progressively reduced use of the resin in favor of other adhesive products. During 1997–2004 prevalence of sensitization was 1.35% including all centers combined, decreasing to 1.21% across the entire study period (1997–2021) excluding Trento/Bolzano/Rovigo.

Sensitization to PTBP-FR was more likely in females, who are generally at higher risk of ACD due to higher level of occupational and non-occupational exposure to allergens in their life-time.

A variability of sensitization was also observed by research center, with lower prevalence in Trento/Bolzano/Rovigo, where agricultural or tourist businesses prevail, compared to Padua and Pordenone, featuring prominent industrial activities. The latter geographical differences may also reflect different environmental conditions, since the provinces of Trentino and Bolzano are mountain areas featured by lower average temperatures than the other three research areas.

## Figures and Tables

**Table 1:** **Prevalence of PTBP-FR sensitization according to main patch-test databases from Europe and North-America, HD = hand dermatitis; LD = leg dermatitis; FD = face dermatitis; ESSCA = European Surveillance System for Contact Dermatitis; IVDK = Information Network of Departments of Dermatology; NAGCD = North American Contact Dermatitis Group**.

Area	Author	Years	Country	N. Patients Tested	% (95% CI)								
					Male Ratio	Occupational Dermatitis	Age > 40	Atopic Dermatitis	HD	LF	FD	PTBP-FR Positivity	
**Europe**	Handley (1993) [9]	1987–1992	Northern Ireland	2270	36.7	-	-	-	-	-	-	0.3	0.89 **
	Marques (1994) [10]	1983–1992	Portugal	3106	44.8	-	-	-	-	67.5 ^$^	-	2.4	
	Geldof (1989) [11]	Before 1989	Netherlands	1966	-	-	-	43.3	-	-	-	1.5	
	Uter (2012) [12]	2007–2008	Austria (ESSCA)	678	34	16	66	17	24	9	14	0.5 (0; 0.9)	
	Uter (2012) [12]	2007–2008	Denmark (ESSCA)	1318	33	24	63	19	43	3	23	1.1 (0.5; 1.7)	
	Uter (2012) [12]	2007–2008	Finland (ESSCA)	760	38 (32; 48)	39 (26; 61)	61 (55; 65)	27 (22; 30)	51 (38; 72)	3 (1; 5)	15 (13; 15)	0.8 (0.1; 1.4)	
	Uter (2012) [12]	2007–2008	Germany (ESSCA)	2712	39 (35; 49)	22 (14; 29)	70 (62; 78)	16 (10; 23)	31 (22; 37)	8 (6; 12)	17 (9; 25)	0.8 (0.4; 1.2)	
	Uter (2012) [12]	2007–2008	Italy (ESSCA)	2938	34 (31; 37)	10 (6; 15)	51 (49; 54)	10 (5; 15)	9 (7; 11)	17 (15; 19)	51 (49; 54)	1.6 (1.2; 2.1)	
	Uter (2012) [12]	2007–2008	Lithuania (ESSCA)	680	26	21	62	17	9	34	62	0.7 (0.1; 1.3)	
	Uter (2012) [12]	2007–2008	Netherlands (ESSCA)	2172	36 (36; 36)	22 (21;24)	56 (53; 58)	27 (25; 30)	24 (12; 30)	5 (4; 5)	21 (17; 26)	1.2 (0.7; 1.6)	
	Uter (2012) [12]	2007–2008	Poland (ESSCA)	793	32 (30; 36)	28 (24; 29)	48 (16; 67)	18 (8; 24)	40 (34; 43)	4 (2; 7)	15 (13; 19)	0.6 (0.1; 1.1)	
	Uter (2012) [12]	2007–2008	Spain (ESSCA)	1845	35 (31; 40)	16 (5; 57)	64 (45; 76)	11 (7; 27)	26 (17; 66)	9 (1; 12)	14 (4; 24)	0.8 (0.3; 1.2)	
	Uter (2012) [12]	2007–2008	Switzerland (ESSCA)	2415	40 (38; 44)	17 (16; 19)	62 (60; 63)	17 (10; 27)	31 (21; 37)	7 (5; 8)	17 (15; 20)	0.8 (0.4; 1.2)	
	Uter (2012) [12]	2007–2008	UK (ESSCA)	9201	33 (27; 39)	11 (8; 18)	57 (51; 64)	-	30 (24; 43)	7 (5; 9)	28 (22; 31)	0.7 (0.5; 0.8)	
	Uter (2020) [13]	2007–2010	Europe (IVDK)	43,052	37.7	15.4	71.9	19.6	27.7	12.0	15.8	0.59 (0.53; 0.67)	
	Uter (2020) [13]	2011–2014	Europe (IVDK)	35,777	36.5	16.9	72.8	23.3	29.9	10.9	16.5	0.50 (0.43; 0.58)	
	Uter (2021) [14]	2015–2018	Europe (ESSCA)	34,453	32.3 *	13.9 *	65.1 *	21.7 *	29.0 *	5.8 *	18.1 *	0.64 (0.58; 0.79)	
	Uter (2022) [15]	2019–2020	Europe (ESSCA)	11,753	30.8 *	13.5 *	61.5 *	22.1 *	25.0 *	6.0 *	17.0 *	0.47 (0.35; 0.61)	
**USA**	Fransway (2013) [16]	2007–2008	USA	5085	35.6	11.8	69.6	21.5	22.8	3.9	25.9	1.2	1.71 **
	Warshaw (2013) [17]	2009–2010	USA	4308	32.1	9.9	67.4	NA	20.1	4.7	15.5	1.5	
	Warshaw (2015) [18]	2011–2012	USA	4235	31.4	9.6	67.3	NA	19.2	3.9	16.1	1.1	
	DeKoven (2017) [19]	2013–2014	USA	4859	30.0	8.9	67.5	NA	20.2	4.7	15.5	1.0	
	DeKoven (2018) [20]	2015–2016	USA	5597	28.0	10.2	67.5	NA	22.0	3.7	16.9	1.2	
	De Koven (2021) [21]	2017–2018	USA	4991	29.5	11.7	64.5	NA	21.5	2.9	15.9	1.1	
	Silverberg (2022) [22]	2001–2018	USA	9966	39.2	18.0	62.2	79.7	22.8	-	-	0.6	
	Silverberg (2022) [22]	2001–2018	USA	1276	53.5	4.0	53.8	-	-	-	2.9	7.1	
	De Koven (2023) [23]	2019–2020	USA	4112	26.2	11.6	64.2	31.8	21.8	3.6	16.0	1.4	
	Zawawi (2023) [24]	2017–2021	USA (Mayo Clinics)	2687	27.8	-	-	-	-	-	-	0.9	

* Median; ** Average %; $ = foot dermatitis. Protective effect (green), harmful effect (yellow).

**Table 2:** **Study population by patch-test results against p-tert-butylphenol-formaldehyde resin (PTBP-FR). Number (N), column and row percentage (%), chi square *p*-value, mean ± standard deviation (M ± SD), median with interquartile range (IQR). CD = contact dermatitis; M = missing values. Obs. = complete case (analysis) observations. ACD = allergic contact dermatitis**.

TERMS			Period 1997–2004 (All Centers)			Period 1997–2021 (Excluding Trento/Bolzano/Rovigo)		
			Total Patients Patch-Tested N (Column %)	PTBP-FR Positivity N (Row % Out of Total Tested)	*p*-Value	Total Patients Patch-Tested N (Column %)	PTBP-FR Positivity N (Row % Out of Total Tested)	*p*-Value
**Total Patients Examined for Suspected ACD**			**15,307**	**206 (1.35)**		**26,996**	**328 (1.21)**	
**1+ positive patch test**	**No**		6743 (44.05)	NA		11,595 (42.95)	NA	
	**Yes**		8464 (55.95)	NA		15,401 (57.05)	NA	
**Center**	**Padua**		5086 (33.23)	90 (1.77)	<0.001	9562 (35.42)	123 (1.29)	0.323
	**Pordenone**		2243 (14.65)	46 (2.05)		7471 (27.67)	97 (1.30)	
	**Trieste**		4345 (28.39)	57 (1.31)		9963 (36.91)	108 (1.08)	
	**Trento/Bolzano/Rovigo**		3633 (23.73)	13 (0.36)		NA	NA	
**Sex**	**Females**		10,323 (67.44)	154 (1.49)	0.024	18,169 (67.30)	251 (1.38)	<0.001
	**Males**		4984 (32.56)	52 (1.04)		8827 (32.70)	77 (0.87)	
**Age** (years) (M: 3)	**M ± SD**		42.7 ± 17.2	42.5 ± 16.3		44.1 ± 17.3	43.1 ± 17.1	
	**Median (IQR)**		40 (29; 55)	40 (30; 53)	0.845 *	42 (30; 57)	41 (29; 55)	0.224 *
	**<41**		7930 (51.81)	102 (1.29)	0.507	12,564 (46.55)	102 (1.29)	0.507
	**>40**		7377 (48.19)	104 (1.41)		14.429 (53.45)	104 (1.41)	
**Atopic dermatitis** (M: 3143)	**No**		11,788 (95.48)	178 (1.51)	0.241	22,754 (89.52)	285 (1.25)	0.372
	**Yes**		558 (4.52)	5 (0.90)		2664 (10.48)	28 (1.05)	
**Occupational dermatitis** (M: 31)	**No**		13,973 (91.29)	192 (1.37)	0.326	24,873 (92.24)	298 (1.20)	0.450
	**Yes**		1334 (8.71)	14 (1.05)		2092 (7.76)	29 (1.39)	
**Body area affected** **by dermatitis**	**Hand** (M: 4432)	**No**	6719 (58.77)	116 (1.73)	0.020	15,045 (65.10)	197 (1.28)	0.490
		**Yes**	4714 (41.23)	56 (1.19)		8528 (34.90)	97 (1.17)	
	**Leg** (M: 4430)	**No**	10,661 (92.81)	159 (1.50)	0.851	21,676 (91.60)	272 (1.25)	0.566
		**Yes**	822 (7.19)	13 (1.58)		1989 (8.40)	22 (1.11)	
	**Face** (M: 4430)	**No**	9585 (83.92)	143 (1.49)	0.778	19,016 (80.35)	245 (1.29)	0.196
		**Yes**	1838 (16.08)	29 (1.58)		4649 (19.65)	49 (1.05)	
**Calendar year**	**1997–2010**		NA	NA	NA	18,371 (68.05)	265 (1.44)	<0.001
	**2011–2021**		NA	NA		8265 (31.95)	63 (0.73)	
**Occupation**	**Clerks**		**3693 (24.13)**	**52 (1.41)**	**0.023**	**5971 (22.12)**	**80 (1.34)**	0.004
	**Health care workers**		**2.133 (13.93)**	**32 (1.50)**		**2572 (9.53)**	**46 (1.79)**	
	**Teachers**		**-**	**-**		**364 (1.35)**	**4 (1.10)**	
	**Cashiers**		**-**	**-**		**26 (0.10)**	**0**	
	**Sellers**		**-**	**-**		**353 (1.31)**	**3 (0.85)**	
	**Restaurant workers**		**631 (4.12)**	**20 (3.17)**		**1118 (4.14)**	**21 (1.88)**	
	**Hairdressers**		**160 (1.05)**	**3 (1.88)**		**330 (1.22)**	**7 (2.12)**	
	**Farmers**		**164 (1.07)**	**1 (0.61)**		**204 (0.76)**	**2 (0.98)**	
	**Construction workers**		**857 (5.60)**	**9 (1.05)**		**944 (3.50)**	**9 (0.95)**	
	**House painters**		**-**	**-**		**26 (0.10)**	**0**	
	**Painter other**		**25 (0.16)**	**0**		**72 (0.27)**	**1 (1.39)**	
	**Construction cleaners**		**-**	**-**		**17 (0.06)**	**1 (5.88)**	
	**Mechanics**		**765 (5.00)**	**5 (0.65)**		**1335 (4.95)**	**13 (0.97)**	
	**Workers of wood industry**		**251 (1.64)**	**2 (0.80)**		**391 (1.45)**	**3 (0.77)**	
	**Artisans general**		**372 (2.43)**	**5 (1.34)**		**346 (1.28)**	**6 (1.73)**	
	**Leather artisans**		**98 (0.64)**	**3 (3.06)**		**90 (0.33)**	**3 (3.33)**	
	**Chemistry Industry workers**		**167 (1.09)**	**4 (2.40)**		**199 (0.74)**	**4 (2.01)**	
	**Drivers**		**191 (1.25)**	**1 (0.52)**		**228 (0.84)**	**1 (0.44)**	
	**General cleaners**		**188 (1.23)**	**4 (2.13)**		**356 (1.32)**	**8 (2.25)**	
	**Housewives**		**2326 (15.20)**	**28 (1.20)**		**2898 (10.73)**	**41 (1.41)**	
	**Students**		**-**	**-**		**830 (3.07)**	**3 (0.36)**	
	**Pensioners**		**2389 (15.61)**	**31 (1.30)**		**3976(14.73)**	**40 (1.01)**	
	**Unemployed**		**229 (1.50)**	**0**		**604 (2.24)**	**3 (0.50)**	
	**Other**		**611 (3.99)**	**6 (0.98)**		**3601 (13.34)**	**29 (0.81)**	
	**Military**		**57 (0.37)**	**0**		**145 (0.54)**	**0**	

* = Wilcoxon test *p*-value.

**Table 3:** Multiple logistic regression analysis on sensitization to p-tert-butyl-phenol-formaldehyde resin (PTBP-FR). Adjusted (aOR9 with 95% confidence interval (95%CI). Obs. = complete case (analysis) observations. **MODEL 1** (adjusted for sex, occupation and center) included all centers but was limited to years 1997–2004. **MODEL 2** (adjusted for sex, occupation and calendar year) stretched across the entire study period (1997–2021) but excluded Trento/Bolzano/Rovigo.

	Terms		All Patients	
			aOR (95%CI)	*p*-Value
**MODEL 1** **Period 1997–2004** (14,996 obs.)	**Center**	**Padua**	reference	
		**Trento/Bolzano/Rovigo**	0.19 (0.11; 0.35)	<0.001
	**Occupation**	**Administrative**	reference	
		**Restaurant workers**	2.44 (1.44; 4.13)	0.001
**MODEL 2** **Period 1997–2021** (excluding Trento/Bolzano/Rovigo) (26,799 obs.)	**Calendar year**	**1997–2010**	reference	
		**2011–2021**	0.52 (0.39; 0.69)	<0.001
	**Sex**	**Females**	reference	
		**Males**	0.69 (0.53; 0.91)	0.008
	**Occupation**	**Administrative**	reference	
		**Other**	0.60 (0.39; 0.91)	0.018

## Data Availability

The original contributions presented in this study are included in the article/Supplementary Materials. Further inquiries can be directed to the corresponding author.

## Supplementary Materials

The following supporting information can be downloaded at: https://www.mdpi.com/article/10.3390/life15050698/s1, Table S1: Triveneto patch-test series (22 haptens) tested in the overall study period (all in pet when not otherwise specified); Table S2: Frequency distribution of patients patch-tested for contact dermatitis and rates of positivity against p-tert-butylphenol-formaldehyde resin (PTBP-FR), by calendar year (1997–2021) and research center. Number (N) and row percentage (%); Table S3: Frequency distribution of patients patch-tested for contact dermatitis and rates of positivity against p-tert-butylphenol-formaldehyde resin (PTBP-FR), by calendar year and research center. Number (N) and row percentage (%).

## References

[1] Adams M.D., Robert M. (1999). Occupational Skin Disease.

[2] Malten K.E., Seutter E. (1985). Allergic degradation products of para-tertiary-butylphenol-formaldehyde plastic. Contact Dermat..

[3] Herro E., Jacob S.E. (2012). P-Tert-Butylphenol Formaldehyde Resin and Its Impact on Children. Dermat. Contact Atopic Occup. Drug.

[4] Traidl S., Werfel T., Ruëff F., Simon D., Lang C., Geier J., Ivdk F.T. (2021). Patch test results in patients with suspected contact allergy to shoes: Retrospective IVDK data analysis 2009–2018. Contact Dermat..

[5] Pereira F., Cunha F. (2000). Allergic Contact Dermatitis from Chromate and 4-Tert-Butylphenol-Formaldehyde Resin in a Father and Daughter. Contact Dermat..

[6] Özkaya E., Elinç-Aslan M.S., Mirzoyeva L. (2010). Allergic Contact Dermatitis Caused by P-Tert-Butylphenol Formaldehyde Resin and Colophonium in Neoprene Thermal Sauna Shorts. Contact Dermat..

[7] Bruynzeel D.P., Andersen K.E., Camarasa J.G., Lachapelle J.M., Menné T., White I.R. (1995). The European standard series. Contact Dermat..

[8] Zimerson E., Bruze B. (2002). Low-molecular-weight contact allergens in p-tert-butylphenol-formaldehyde resin. Am. J. Contact Dermat..

[9] Handley J.D., Bingham T.A., Corbett R., Burrows D. (1993). Allergic Contact Dermatitis from Para-Tertiary Butylphenol-Formaldehyde Resin (PTBP-F-R) in Northern Ireland. Contact Dermat..

[10] Marques C., Gonzalo M., Gonzalo S. (1994). Sensitivity to para-tertiary-butylphenol-formaldehyde resin in Portugal. Contact Dermat..

[11] Geldof B.A., Roesyanto I.D., van Joost T. (1989). Clinical Aspects of Para-Tertiary-Butylphenol-formaldehyde Resin (PTBP-FR) Allergy. Contact Dermat..

[12] Uter W., Aberer W., Armario-Hita J.C., Fernandez-Vozmediano J.M., Ayala F., Balato A., Bauer A., Ballmer-Weber B., Beliauskiene A., Fortina A.B. (2012). Current patch test results with the European baseline series and extensions to it from the ‘European Surveillance System on Contact Allergy’ network, 2007–2008. Contact Dermat..

[13] Uter W., Gefeller O., Mahler V., Geier J. (2020). Trends and current spectrum of contact allergy in Central Europe: Results of the Information Network of Departments of Dermatology (IVDK) 2007–2018. Br. J. Dermatol..

[14] Uter W., Bauer A., Belloni Fortina A., Bircher A.J., Brans R., Buhl T., Cooper S.M., Czarnecka-Operacz M., Dickel H., Dugonik A. (2021). Patch test results with the European baseline series and additions thereof in the ESSCA network, 2015–2018. Contact Dermat..

[15] Uter W., Wilkinson S.M., Aerts O., Bauer A., Borrego L., Brans R., Buhl T., Dickel H., Dugonik A., Filon F.L. (2022). Patch test results with the European baseline series, 2019/20-Joint European results of the ESSCA and the EBS working groups of the ESCD, and the GEIDAC. Contact Dermat..

[16] Fransway A.F., Zug K.A., Belsito D.V., DeLeo V.A., Fowler J.F., Maibach H.I., Marks J.G., Mathias C.T., Pratt M.D., Rietschel R.L. (2013). North American Contact Dermatitis Group patch test results for 2007–2008. Dermatitis.

[17] Warshaw E.M., Belsito D.V., Taylor J.S., Sasseville D., DeKoven J.G., Zirwas M.J., Fransway A.F., Mathias C.G.T., Zug K.A., DeLeo V.A. (2013). North American Contact Dermatitis Group patch test results: 2009 to 2010. Dermatitis.

[18] Warshaw E.M., Maibach H.I., Taylor J.S., Sasseville D., DeKoven J.G., Zirwas M.J., Fransway A.F., Mathias C.G.T., Zug K.A., DeLeo V.A. (2015). North American contact dermatitis group patch test results: 2011–2012. Dermatitis.

[19] DeKoven J.G., Warshaw E.M., Belsito D.V., Sasseville D., Maibach H.I., Taylor J.S., Marks J.G., Fowler J.F., Mathias C.G., DeLeo V.A. (2017). North American Contact Dermatitis Group Patch Test Results 2013–2014. Dermatitis.

[20] DeKoven J.G., Warshaw E.M., Zug K.A., Maibach H.I., Belsito D.V., Sasseville D., Taylor J.S., Fowler J.F., Mathias C.G.T., Marks J.G. (2018). North American Contact Dermatitis Group Patch Test Results: 2015–2016. Dermatitis.

[21] DeKoven J.G., Silverberg J.I., Warshaw E.M., Atwater A.R., Reeder M.J., Sasseville D., Taylor J.S., Zug K.A., Belsito D.V., Maibach H.I. (2021). North American Contact Dermatitis Group Patch Test Results: 2017–2018. Dermatitis.

[22] Silverberg J.I., Patel N., Warshaw E.M., DeKoven J.G., Belsito D.V., Atwater A.R., Houle M.-C., Taylor J.S., Reeder M.J., Zug K.A. (2022). Hand and foot dermatitis in patients referred for patch testing: Analysis of North American Contact Dermatitis Group Data, 2001–2018. J. Am. Acad. Dermatol..

[23] DeKoven J.G., Warshaw E.M., Reeder M.J., Atwater A.R., Silverberg J.I., Belsito D.V., Sasseville D., Zug K.A., Taylor J.S., Pratt M.D. (2023). North American Contact Dermatitis Group Patch Test Results: 2019–2020. Dermatitis.

[24] Zawawi S., Yang Y.W., Cantwell H.M., Drage L.A., Youssef M.J., Yiannias J.A., Davis M.D.P., Hall M.R. (2023). Trends in Patch Testing With the Mayo Clinic Standard Series, 2017–2021. Dermatitis.

[25] Zahida R., Hussain I., Saeed Haroon T. (2003). Common Allergens in Shoe Dermatitis: Our Experience in Lahore, Pakistan. Int. J. Dermatol..

[26] Pinar O., Polat M., Cinar L., Alli N. (2007). Shoe Dermatitis from Para-Tertiary Butylphenol Formaldehyde. Contact Dermat..

[27] Warshaw E.M., Schram S.E., Belsito D.V., DeLeo V.A., Fowler J.F., Maibach H.I., Marks J.G., Mathias T.C., Pratt M.D., Rietschel R.L. (2007). Shoe allergens: Retrospective analysis of cross-sectional data from the north American contact dermatitis group, 2001–2004. Dermatitis.

[28] Atwater A.R., Bembry R., Green C.L., DeKoven J.G., Warshaw E.M., Belsito D.V., Maibach H.I., Silverberg J.I., Taylor J.S., Reeder M.J. (2022). Shoe Allergens: A Retrospective Analysis of Cross-Sectional Data From the North American Contact Dermatitis Group, 2005–2018. Dermatitis.

[29] Chowdhuri S., Ghosh S. (2007). Epidemio-Allergological Study in 155 Cases of Footwear Dermatitis. Indian J. Dermatol. Venereol. Leprol..

[30] Mancuso G., Reggiani M., Berdondini R.M. (1996). Occupational Dermatitis in Shoemakers. Contact Dermat..

[31] Chen Y.X., Gao B.A., Cheng H.Y., Li L.F. (2017). Survey of Occupational Allergic Contact Dermatitis and Patch Test among Clothing Employees in Beijing. BioMed Res. Int..

[32] Bruze M., Almgren G. (1988). Occupational dermatoses in workers exposed to resins based on phenol and formaldehyde. Contact Dermat..

[33] Mathys E., Zahir A., Ehrlich A. (2014). Shoe Allergic Contact Dermatitis. Dermatitis.

[34] Avenel-Audran M., Goossens A., Zimerson E., Bruze M. (2003). Contact dermatitis from electrocardiograph monitoring electrodes: Role of p-tert-butylphenol-formaldehyde resin. Contact Dermat..

[35] Kellogg C.C., Choi A.W., Shaw D.W. (2022). Allergic Contact Dermatitis to p-tert-Butylphenol-Formaldehyde Resin From the Label Adhesive of an Electrocardiogram Electrode. Dermatitis.

[36] Corazza M., Bencivelli D., Catani M., Cavazzini A., Mantovani L., Borghi A. (2021). Occupational allergic contact dermatitis in a basket player due to phenolic resins. Contact Dermat..

[37] Nagashima C., Tomitaka-Yagami A., Matsunaga K. (2003). Contact dermatitis due to para-tertiary-butylphenol363 formaldehyde resin in a wetsuit. Contact Dermat..

[38] Uter W., Gefeller O., Geier J., Schnuch A. (2008). Changes of the patch test population (MOAHLFA index) in long term participants of the Information Network of Departments of Dermatology, 1999–2006. Contact Dermat..

[39] Johansen J.D., Aalto-Korte K., Agner T., Andersen K.E., Bircher A., Bruze M., Cannavó A., Giménez-Arnau A., Gonçalo M., Goossens A. (2015). European Society of Contact Dermatitis guideline for diagnostic patch testing—Recommendations on best practice. Contact Dermat..

[40] Japundžić I., Novak-Hlebar I., Špiljak B., Kuna M., Yale K., Lugović-Mihić L. (2022). Skin Features Important For The Occurrence Of Contact Dermatitis In Healthcare Workers. Acta Clin. Croat..

[41] Fluhr J.W., Akengin A., Bornkessel A., Fuchs S., Praessler J., Norgauer J., Grieshaber R., Kleesz P., Elsner P. (2005). Additive impairment of the barrier function by mechanical irritation, occlusion and sodium lauryl sulphate in vivo. Br. J. Dermatol..

[42] Zhai H., Maibach H.I. (2001). Skin occlusion and irritant and allergic contact dermatitis: An overview. Contact Dermat..

[43] Fluhr J.W., Praessler J., Akengin A., Fuchs S.M., Kleesz P., Grieshaber R., Elsner P. (2005). Air flow at different temperatures increases sodium lauryl sulphate-induced barrier disruption and irritation in vivo. Br. J. Dermatol..

[44] John S.M., Uter W. (2005). Meteorological influence on NaOH irritation varies with body site. Arch. Dermatol. Res..

[45] ECHA P-TERT-BUTYLPHENOL CAS No: 98-54-4 EINECS No–ECHA. https://echa.europa.eu/documents/10162/296c0932-cf7a-4d90-9051-ba7b224a32b3.

[46] Smith V.M., Wilkinson S.M. (2015). A multicentre audit of cutaneous allergy (patch testing) services within Yorkshire, UK. Clin. Exp. Dermatol..

[47] Thyssen Pontoppidan J., Linneberg A., Menné T., Johansen J.D. (2007). The Epidemiology of Contact Allergy in the General Population –Prevalence and Main Findings. Contact Dermat..

[48] Hamann C.R., Zimerson E., Hamann D., Laugesen L., Carlsson B., Nathansen C., Hamann C., Bruze M. (2012). Concentration Variability of Potent Allergens of P-Tert-Butylphenol-Formaldehyde Resin (PTBP-FR) in Patch Test Preparations and Commercially Available PTBP-FR. Br. J. Dermatol..

[49] Lintu P., Soramaki L.S., Liippo J. (2020). Clinical Relevance of P-Tert-Butylphenol-Formaldehyde Resin (PTBP408 FR) Contact Allergy among General Dermatology Patients. Contact Dermat..

[50] Mauro M., Bovenzi M., Laese Filon F. (2021). Perspective: North East Italian Data 1996–2016: OCD in a Gender Perspective in North Italy. Med. Lav..

[51] Santarossa M., Mauro M., Belloni Fortina A., Corradin M.T., Larese Filon F. (2020). Occupational contact dermatitis in Triveneto: Analysis of patch test data of the North Eastern Italian Database from 1996 to 2016. Contact Dermat..

[52] Massone L., Anonide A., Borghi S., Usiglio D. (1996). Sensitization to Para-Tertiary-Butylphenolformaldehyde Resin. Int. J. Dermatol..

[53] Uter W., Gefeller O., Giménez-Arnau A., Frosch P., Johansen J.D., Schuttelaar M.L., Rustemeyer T., Filon F.L., Dugonik A., Bircher A. (2015). Characteristics of Patients Patch Tested in the European Surveillance System on Contact Allergies (ESSCA) Network, 2009–2012: ESSCA 2009–2012. Contact Dermat..

[54] Alinaghi F., Bennike N., Egeberg A., Thyssen J., Johansen J. (2019). Prevalence of contact allergy in the general population: A systematic review and meta-analysis. Contact Dermat..

[55] ESSCA Writing Group (2008). The European Surveillance System of Contact Allergies (ESSCA): Results of patch testing the standard series, 2004. J. Eur. Acad. Dermatol. Venereol..

[56] Uter W., Amario-Hita J.C., Balato A., Ballmer-Weber B., Bauer A., Fortina A.B., Bircher A., Chowdhury M., Cooper S., Czarnecka-Operacz M. (2017). European Surveillance System on Contact Allergies (ESSCA): Results with the European baseline series, 2013/14. J. Eur. Acad. Dermatol. Venereol..

[57] Martellotta D., Di Costanzo L., Cafiero M., La Bella S., Balato A. (2008). Contact allergy to p-tert-butylphenol formaldehyde resin and zinc diethyldithiocarbamate in a wet suit. Dermatitis.

[58] Foussereau J., Cavelier C., Selig D. (1976). Occupational eczema from para-tertiary-butylphenol formaldehyde resins: A review of the sensitizing resins. Contact Dermat..

[59] Cegolon L., Larese Filon F., on behalf of the North-East Research Group on Contact Dermatitis (2024). Sensitization to Lanolin in North-Eastern Italy, 1997–2021: Prevalence, Risk Factors and the Impact of Occupation. Contact Dermat. Life.

[60] Cegolon L., Larese Filon F. (2025). Prevalence and determinants of sensitization to neomycin in North-Eastern Italy, 1997–2021. Contact Dermat..

