# Supplementary material for: Epidemiological and Occupational Pattern of Patch-Test Reactions to p-Tert-butylphenol-formaldehyde Resin in North-Eastern Italy, 1997–2021
Source: Life (Basel). 2025 Apr 25;15(5):698. doi: 10.3390/life15050698 (PMC12113552; doi:10.3390/life15050698)
Supplement: Supplementary file 1 [file life-15-00698-s001.zip › life-3570463-supplementary.pdf]

Table S1. Triveneto patch-test series (22 haptens) tested in the overall study period (all in pet when not otherwise specified).

1	4-ter-Butylphenol- formaldehyde resin 1%
2	Carba mix 3%
3	Cobalt chloride hexahydrate 1%
4	Colophonium 20%
5	Disperse blu 35 1%
6	Disperse yellow 3 1%
7	Epoxy resin 1%
8	Formaldehyde 1% aq
9	Fragrance mix-I 8%
10	Methyl-chloro-isothiazolinon/methyl-isothiazolinon (Kathon) 0.02 aq.
11	Lanolin alcohol 30%,
12	Mercaptobenzothiazole 2%
13	Mercaptobenzothiazole mix 2%
14	Neomycin sulfate 20%
15	Nickel sulphate 5%
16	N-Isopropy-N-phenyl-4-phenylendiamine 0.1%
17	Parabens mix 16%
18	Peru balsam 25%
19	Potassium bichromate 0.5%
20	p-Phenylenediamine 1%
21	Quaternium-15 1%
22	Thiuram mix 1%

Table S2. Frequency distribution of patients patch-tested for contact dermatitis and rates of positivity against p-tert-butylphenol-formaldehyde resin (PTBP-FR), by calendar year (1997-2021) and research center. Number (N) and row percentage (%).

CALENDAR YEAR	ENTIRE COHORT		RESEARCH CENTER							
			Padua		Pordenone		Trieste		Trento/Bol-zano/Rovigo	
	N. tests	PTBP-FR+	N. tests	PTBP-FR+	N. tests	PTBP-FR+	N. tests	PTBP-FR+	N. tests	PTBP-FR+
**1997**	1,242	13 (1.05)	395	3 (0.76)	445	8 (1.80)	398	2 (0.50)	4	0
**1998**	2,566	27 (1.05)	1,083	13 (1.20)	325	6 (1.85)	418	4 (0.96)	740	4 (0.54)
**1999**	2,868	40 (1.39)	1,189	21 (1.77)	278	7 (2.52)	453	4 (0.88)	948	8 (0.84)
**2000**	2,445	48 (1.96)	1,042	24 (2.30)	208	4 (1.92)	817	20 (2.45)	378	0
**2001**	2,412	26 (1.08)	677	15 (2.22)	226	2 (0.88)	703	8 (1.14)	806	1 (0.12)
**2002**	1,460	19 (1.30)	1	0	353	8 (2.27)	701	11 (1.57)	405	0
**2003**	1,472	22 (1.49)	412	8 (1.94)	314	9 (2.87)	485	5 (1.03)	261	0
**2004**	842	11 (1.31)	287	6 (2.09)	94	2 (2.13)	370	3 (0.81)	91	0
**2005**	1,135	10 (0.88)	434	2 (0.46)	383	6 (1.57)	318	2 (0.63)	0	NA
**2006**	1,032	15 (1.45)	409	3 (0.73)	336	10 (2.98)	287	2 (0.70)	0	NA
**2007**	1,115	12 (1.08)	439	5 (1.14)	368	3 (1.13)	308	4 (1.30)	0	NA
**2008**	1,257	15 (1.19)	528	4 (0.76)	355	4 (1.13)	374	7 (1.87)	0	NA
**2009**	1,112	13 (1.17)	328	2 (0.61)	373	4 (1.07)	411	7 (1.70)	0	NA
**2010**	1,046	7 (0.67)	354	2 (0.56)	366	3 (0.82)	326	2 (0.61)	0	NA
**2011**	1,107	14 (1.26)	234	2 (0.85)	377	7 (1.86)	496	5 (1.01)	0	NA
**2012**	664	6 (0.90)	0	2 (0.85)	286	4 (1.40)	378	2 (0.53)	0	NA
**2013**	886	3 (0.34)	240	2 (0.83)	274	0	372	1 (0.27)	0	NA
**2014**	1,011	7 (0.69)	322	4 (1.24)	288	1 (0.35)	401	2 (0.50)	0	NA
**2015**	958	9 (0.94)	246	3 (1.22)	327	1 (0.31)	385	5 (1.30)	0	NA
**2016**	749	2 (0.27)	230	0	239	0	280	2 (0.71)	0	NA
**2017**	501	4 (0.80)	102	3 (2.94)	241	0	158	1 (0.63)	0	NA
**2018**	836	10 (1.20)	135	0	451	5 (1.11)	250	5 (2.00)	0	NA
**2019**	1,008	3 (0.30)	381	1 (0.26)	211	0	416	2 (0.48)	0	NA
**2020**	540	3 (0.56)	94	0	265	3 (1.13)	181	0	0	NA
**2021**	365	2 (0.55)	0	-	88	0	277	2 (0.72)	0	NA
**Total**	**30,629**	**341 (1.11)**	**9,562**	**123 (1.29)**	**7,471**	**97 (1.30)**	**9,963**	**108 (1.08)**	**3,633**	**13 (0.36)**

Supplementary Table S3. Frequency distribution of patients patch-tested for contact dermatitis and rates of positivity against p-tert-butylphenol-formaldehyde resin (PTBP-FR), by calendar year and research center. Number (N) and row percentage (%).

CALENDAR YEAR	ENTIRE COHORT		RESEARCH CENTER							
			Padua		Pordenone		Trieste		Trento/Bolzano/Rovigo	
	N. tests	PTBP-FR+	N. tests	PTBP-FR+	N. tests	PTBP-FR+	N. tests	PTBP-FR+	N. tests	PTBP-FR+
**1997-2004**	15,307	206 (1.30)	5,086	90 (1.77)	2,243	66 (2.05)	4,345	57 (1.31)	3,633	13 (0.36)
**2005-2021**	15,322	135 (0.88)	4,476	33 (0.74)	5,228	51 (0.98)	5,618	51 (0.91)	-	-
**1997-2010***	18,371	265 81.44)	7,578	108 (1.43)	4,424	76 (1.72)	6,369	81 (1.27)	-	-
**2011-2021**	8,625	63 (0.73)	1,984	15 (0.76)	3,047	21 (0.69)	3,594	27 (0.75)	-	-

* Excluding Trento/Bolzano/Rovigo.
